# Multimodal MRI-based radiomic nomogram for predicting telomerase reverse transcriptase promoter mutation in IDH-wildtype histological lower-grade gliomas

**DOI:** 10.1097/MD.0000000000036581

**Published:** 2023-12-22

**Authors:** Xulei Huo, Yali Wang, Sihan Ma, Sipeng Zhu, Ke Wang, Qiang Ji, Feng Chen, Liang Wang, Zhen Wu, Wenbin Li

**Affiliations:** a Department of Neurosurgery, Beijing Tiantan Hospital, Capital Medical University, Beijing, China; b Department of Neuro-oncology, Cancer Center, Beijing Tiantan Hospital, Capital Medical University, Beijing, China.

**Keywords:** glioma, IDH wildtype, nomogram, radiomic, TERTp mutation

## Abstract

The presence of TERTp mutation in isocitrate dehydrogenase-wildtype (IDHwt) histologically lower-grade glioma (LGA) has been linked to a poor prognosis. In this study, we aimed to develop and validate a radiomic nomogram based on multimodal MRI for predicting TERTp mutations in IDHwt LGA. One hundred and nine IDH wildtype glioma patients (TERTp-mutant, 78; TERTp-wildtype, 31) with clinical, radiomic, and molecular information were collected and randomly divided into training and validation set. Clinical model, fusion radiomic model, and combined radiomic nomogram were constructed for the discrimination. Radiomic features were screened with 3 algorithms (Wilcoxon rank sum test, elastic net, and the recursive feature elimination) and the clinical characteristics of combined radiomic nomogram were screened by the Akaike information criterion. Finally, receiver operating characteristic curve, calibration curve, Hosmer–Lemeshow test, and decision curve analysis were utilized to assess these models. Fusion radiomic model with 4 radiomic features achieved an area under the curve value of 0.876 and 0.845 in the training and validation set. And, the combined radiomic nomogram achieved area under the curve value of 0.897 (training set) and 0.882 (validation set). Above that, calibration curve and Hosmer–Lemeshow test showed that the radiomic model and combined radiomic nomogram had good agreement between observations and predictions in the training set and the validation set. Finally, the decision curve analysis revealed that the 2 models had good clinical usefulness for the prediction of TERTp mutation status in IDHwt LGA. The combined radiomics nomogram performed great performance and high sensitivity in prediction of TERTp mutation status in IDHwt LGA, and has good clinical application.

## 1. Introduction

The recent 2021 World Health Organization (WHO) classification of gliomas highlights the growing importance of molecular diagnostics, particularly in the case of isocitrate dehydrogenase-wildtype (IDHwt) diffuse gliomas.^[1]^ A lower-grade astrocytoma (lower-grade glioma [LGA]; WHO grade 2–3) with an IDHwt histology and telomerase reverse transcriptase promoter (TERTp) mutation is now recognized as having a poor clinical prognosis^[2–4]^ and considered a glioblastoma, IDHwt according to this classification.^[1,5]^ The 2021 WHO classification emphasizes the significance of molecular features in the prognosis of IDHwt histological LGAs and molecular diagnostic testing of these parameters is now a requirement. However, the potential role of imaging biomarkers in predicting prognosis for this type of glioma has not yet been fully explored.

Radiomics, a quantitative imaging analysis method, has emerged as a promising tool for noninvasively characterizing tumors by extracting high-dimensional imaging features.^[6]^ While recent studies have demonstrated the potential of radiomics in predicting molecular features of neuro system tumors, such as meningiomas,^[7]^ pineal region tumors,^[8]^ and gliomas with IDH mutations,^[9–12]^ the role of radiomics in predicting TERTp-mutation in IDHwt LGAs has not been fully explored. Therefore, the aim of this study was to develop machine learning models that utilize radiomics features extracted from multi-parameter MRI images to predict TERTp-mutation in IDHwt LGAs. By constructing a combined Radiomics + Clinical nomogram, we sought to analyze and predict TERTp-mutation status in individual patients. Successful development of such a model could facilitate noninvasive identification of TERTp-mutation and contribute to personalized treatment, ultimately improving patient outcomes.

## 2. Methods

This study was approved by the ethics committee of Beijing tiantan hospital, and the patients or legal guardians waived their rights to consent in the retrospective study.

### 2.1. Patient enrollment

Total 109 IDHwt LGAs after surgery from January 2021 to December 2022 were collected in the study. All patients were randomly divided into the training set (n = 78, model construction) and validation set (n = 31, model validation). The inclusion criteria were as follows: (I) IDHwt LGAs confirmed by histopathology defined as infiltrative astrocytoma lacking histopathologic features of glioblastoma (microvascular proliferation and/or necrosis), (II) patients with known TERTp mutation status and IDH wildtype status, (III) diagnosis and initial tumor resection performed at our hospital, and (IV) preoperative MRI scans, including T2-weighted imaging (T2WI) and contrast-enhanced T1-weighted imaging (CE-T1WI), recorded in a Picture Archiving and Communication System. The exclusion criteria were (a) lack of clinical information or incomplete MRI sequences; (b) age < 18 years; (c) an error in image processing; and (d) unknown TERTp mutation status. IDH 1/2 mutation and TERTp mutation were done by the Sanger sequence and next generation sequence in the Institutional pathology department.

Eleven clinical and molecular information of the enrolled patients were follows: WHO grade, age, gender, midline shift (Yes or No), laterality (Unilateral or Bilateral), Peritumoral edema (Yes or No), Enhancing margin (Well defined or Poorly defined), Enhancement style (Irregular-enhancement, Nodular-enhancement, Ring-enhancement, and No), Enhancement extent (NO, slight, and obvious), and MGMT methylation status (Yes and No).

### 2.2. Brain MRI sequence

**Figure 1.**
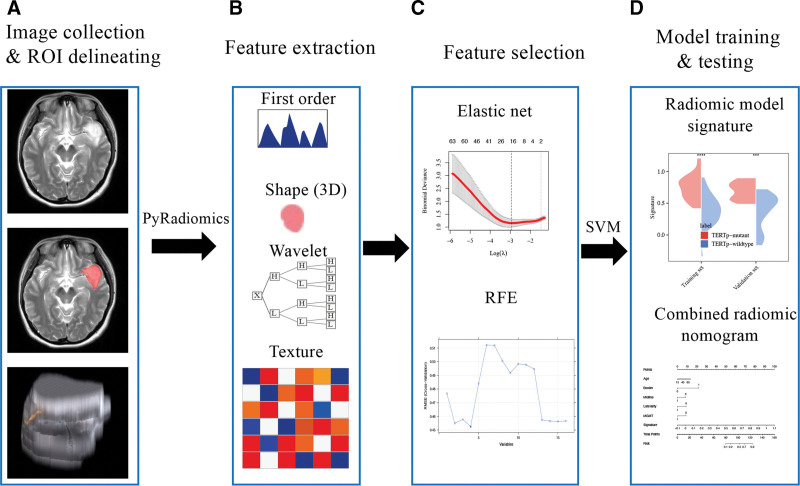
The workflow of the study. (A) Brain MR images and ROI delineating. (B) Four classes radiomics features extraction (Pyradiomics algorithm). (C) Feature selection with elastic net and RFE algorithm. (D) Radiomic model and combined radiomic nomogram.

Figure 1 showed the workflow of this study. The axial T2WI and axial CE-T1WI were used in the study. Before the examination, all patients signed the informed consent and removed all metal objects. And, the examination was done on the spine position with the 3.0-T scanner (Siemens Magnetom Skyra, Malvern, PA). For T2WI and T1WI repetition time was 4900 ms and 1770 ms; echo time was 117.12 ms and 9.4 ms; acquisition matrix was 320 × 288 and 256 × 198; flip angle was 90° and 150°, respectively. In addition, field of view, slice thickness, and spacing between slices were both 100 × 100, 5 mm, and 6 mm. Furthermore, The CE-T1WI was done after the patients received the injection of the contrast agent (gadolinium-DTPA, Magnevist, 0.1 mmol/kg) with the parameter of T1WI. These images were stored as the DICOM format on the picture archiving and communication system.

### 2.3. Regions of interest delineating

Three dimensional regions of interest (ROI) delineating was done by a neuroradiologist with 8 years of experience with the help of MRIcron (http://www.mricro.com; University of South Carolina, Columbia, SC) according to the edge of the tumor. Then, another neuroradiologist with 10 years of experience confirmed the results. Any disagreement between the 2 neuroradiologists will be solved by the neuroradiologist with 25 years of experience.

### 2.4. Radiomic feature extraction

PyRadiomics (version 2.1.2) was used to extract radiomic properties from ROIs. The detailed algorithms and feature explanations can be found at https://github.com/Radiomics/pyradiomics.^[13]^ Gray values were discretized using a fixed bin width. The features of T2WI and CE-T1WI were extracted, respectively. Total 1304 radiomic features were extracted from each ROI with 6 built-in filters including wavelet, Laplacian of Gaussian, square, square root, logarithm, and exponential. Finally, the features could be assigned into 4 categories that 14 first order statistics, 126 shape features, 688 wavelet features, and 476 texture classes.^[14]^

In addition, the shape features primarily describe the size and shape of the tumor region in 3 dimensions; first-order statistic describes the distribution of voxel intensities within the image region defined by the mask with commonly used and basic metrics; texture features describing patterns or the spatial distribution of voxel intensities, which has 4 types that were gray level dependence matrix, gray level co-occurrence matrix, gray level run length matrix, and gray level size zone; wavelet transform effectively separates textural information from high- and low-frequency components of the original image, which is similar with the Fourier transform analysis.

### 2.5. Radiomic features selection

All feature values were normalized based on minimum–maximum normalization with the following formula, and the Xmin and Xmin was the respective minimum and maximum values of the feature Xn:


$$\begin{array}{l} Normalized\;\;\;Xn=\frac{Xn-Xmin}{Xmax-Xn} \end{array}$$

Based on these normalized features, features were selected and models were trained. Moreover, since the radiomic features are numerous and complex, we needed to perform a selection process to reduce overfitting. On the training set, the selection process was conducted as described previously.^[8]^ Three methods was used to prioritize the features: (I) hypothesis test with the Wilcoxon rank sum test, which retained the characteristics that were different between the 2 categories (*P* < .05)^[14]^; (II) the elastic net approach^[15]^ was used to select the most insightful features. Elastic net was a selection operator combining least absolute shrinkage and selection operator and ridge regression. With tuning parameter alpha (0–1, step 0.1), the E-net was trained, and the λ was confirmed with 10-fold cross-validation, which followed the minimum standard deviation criteria. A model with final alpha and λ values was used to select features with nonzero coefficients; (III) the radiomic features were subsequently identified by recursive feature elimination (RFE) through 5-fold validation.^[8]^ RFE was proposed by Guyon et al.^[16]^ The goal is to rank the features and recursively remove the ones that contribute least to classification. Weights are assigned to the features in order to train the estimator. The smallest weight features will be pruned from the current set of features, and the procedure will be repeated until the desired number of features is reached.

### 2.6. Fusion radiomic model construction

Final selected radiomic features were used to create a fusion radiomic model through the support vector machine (SVM) algorithm. SVM creates a high-dimensional hyperplane in which different classes are separated.^[17]^ Larger functional margin represents for better separation by the hyperplane, which means the generalization error is lower. Both regression and classification tasks could be realized through SVM. The parameters determined by GridSearch method are those had the best performance in the model. Furthermore, fusion radiomic model differences between TERTp-mutant and TERTp-wildtype patients in IDHwt LGAs were compared with a violin plot.

### 2.7. Construction of combined radiomics nomogram

Multivariable logistic regression analysis was used to construct a clinical model based on the clinical features. And, the Akaike information criterion (AIC) was used to select the most valuable clinical features. Furthermore, for establishing a more accurate and comprehensive model for the tumor discrimination, multivariable logistic regression was used to construct a combined radiomic nomogram with the above selected clinical features and the fusion radiomic model. A nomogram was created to illustrate the structure and parameters of the combined radiomic model with “rms” package (version 6.3.0).

### 2.8. The discrimination analysis

To evaluate the discriminative efficacy of the clinical model, fusion model, combined radiomic nomogram, the receiver operating characteristic (ROC) curve was done on the training and validation sets with the “pROC” package. And the area under the ROC curve (AUC), accuracy (ACC), sensitivity, positive predict value, and negative predictive value were revealed from the ROC curve. To assess the similarity between observed results and predicted diagnosis results of the fusion radiomic model and combined radiomic nomogram, calibration curves and the Hosmer–Lemeshow test were performed. Based on the DCA method with “rmda” package (version 1.6), the net benefits at different threshold probabilities were quantified for the fusion model and combined radiomics nomogram. Finally, the De long test was performed to compare the statistical significance of the AUC value between the clinical model, fusion radiomic model, and the combined radiomic nomogram.

### 2.9. Statistical analysis

Two-sided *P*-value < .05 was considered statistically significant. All analyses were carried out on R (version 4.2.1, R Foundation for Statistical Computing, Vienna). Chi-Square test was used to compare the differences in categorical variables, while nonparametric test was used to compare the differences of continuous variables that do not conform to the normal distribution.

## 3. Results

### 3.1. Clinical characteristics

In the study, 109 IDH wildtype glioma patients with a mean age of 49.5 (13.9) years were collected according to the inclusion and exclusion criteria. The gender ratio (male/female) of the study was 1.6:1 (67/42). And, the tumor location of 19 (17.4%) patients had involved the midline. 63 (57.8%) patients have TERTp-mutant status and 46 (42.2%) patients have TERTp-wildtype status. All clinical information were showed in Table 1 and Table S1 (Supplemental Digital Content, http://links.lww.com/MD/L63, which illustrates the detailed information of all patients). As shown in Table 1, age showed significant relationships in IDHwt LGAs (*P* < .01) between TERTp-mutant and TERTp-wildtype, which revealed that younger patients will be diagnosis as TERTp-wildtype. In contrast, there was no significant difference in gender (*P* = .773), midline (*P* = .311), laterality (*P* = .276), peritumoral edema (*P* = .236), Enhancing margin (*P* = .307), Enhancement style (*P* = .203), Enhancement extent (*P* = .269), and MGMT methylation status (*P* = .508) between the 2 groups.

**Table 1:** Clinical characteristics of IDH wildtype patients.

Characteristic	All patients (n = 109)	TERTp-mutant (n = 63)	TERTp-wildtype (n = 46)	*P* value
*Gender*				
Male	67 (61.5%)	38 (60%)	29 (63%)	.773
Female	42 (38.5%)	25 (40%)	17 (37%)	
Age (year)	49.5 (13.9)	53.3 (11.4)	44.2 (15.2)	.002
*Midline*				
Involving	19 (17.4%)	9 (14.3%)	10 (21.7%)	.311
Not involving	90 (82.6%)	54 (85.7%)	36 (78.3%)	
*Laterality*				
Unilateral	86 (78.9%)	52 (82.5%)	34 (73.9%)	.276
Bilateral	23 (21.1%)	11 (17.5%)	12 (26.1%)	
*Peritumoral edema*				
Yes	64 (58.7%)	40 (63.5%)	24 (52.2%)	.236
No	45 (41.3%)	23 (36.5%)	22 (47.8%)	
*Enhancing margin*				
Well defined	53 (48.6)	28 (44.4%)	25 (54.3%)	.307
Poorly defined	56 (51.4%)	35 (55.6%)	21 (45.7%)	
*Enhancement style*				
No	32 (29.4%)	22 (34.9%)	10 (21.7%)	.203
Irregular-enhancement	43 (39.5)	24 (38.1%)	19 (41.3%)	
Ring-enhancement	22 (20.2%)	13 (20.6%)	9 (19.6%)	
Nodular-enhancement	12 (11.0%)	4 (6.3%)	8 (17.4%)	
*Enhancement extent*				
No	32 (29.4%)	22 (34.9%)	10 (21.7%)	.269
Slight	35 (32.1%)	20 (31.7%)	15 (32.6%)	
Obvious	42 (38.5%)	21 (33.3%)	21 (39.1%)	
*^M^MGMT status*				
Yes	37 (33.9%)	23 (36.5%)	14 (30%)	.508
No	72 (66.1%)	40 (63.5%)	32 (70%)	

*Note*: Categorical variables were presented as the number (percentage). Age was presented as mean ± standard deviation.

M MGMT status = MGMT methylation status.

All patients were assigned to training set (n = 78) and validation set (n = 31) (Tables 2 and 3). And, there was no significant difference among the clinical information including gender (*P* = .981), age (*P* = .211), midline (*P* = .739), laterality (*P* = .811), peritumoral edema (*P* = .342), Enhancing margin (*P* = .095), Enhancement style (*P* = .223), Enhancement extent (*P* = .073), and MGMT methylation status (*P* = .508) between training set and the validation set. The training set and the validation set justify the unbalance of TERT*p*-mutant status of the IDHwt LGAs.

**Table 2:** Clinical characteristics of training and validation sets.

Characteristic	All sets (n = 109)	Training set (n = 78)	Validation set (n = 31)	*P*-value
*Gender*				
Male	67 (61.5%)	48 (61.5%)	19 (61.3%)	.981
Female	42 (38.5%)	30 (38.5%)	12 (38.7%)	
Age (year)	49.5 (13.9)	50.3 (14.1)	47.4 (13.2)	.211
*Midline*				
Involving	19 (17.4%)	13 (16.7%)	6 (19.4%)	.739
Not involving	90 (82.6%)	65 (83.3%)	25 (80.7%)	
*Laterality*				
Unilateral	86 (78.9%)	62 (79.5%)	24 (77.4%)	.811
Bilateral	23 (21.1%)	16 (20.5%)	7 (22.6%)	
*Peritumoral edema*				
Yes	64 (58.7%)	48 (61.5%)	16 (51.6%)	.342
No	45 (41.3%)	30 (38.5%)	15 (48.4%)	
*Enhancing margin*				
Well defined	53 (48.6)	34 (43.6%)	19 (61.3%)	.095
Poorly defined	56 (51.4%)	44 (56.4%)	12 (38.7%)	
*Enhancement style*				
No	32 (29.4%)	20 (25.6%)	12 (38.7%)	.223
Irregular-enhancement	43 (39.5)	35 (44.9%)	8 (25.8%)	
Ring-enhancement	22 (20.2%)	16 (20.5%)	6 (19.4%)	
Nodular-enhancement	12 (11.0%)	7 (9.0%)	5 (16.1%)	
*Enhancement extent*				
No	32 (29.4%)	20 (25.6%)	12 (38.7%)	.073
Slight	35 (32.1%)	30 (38.5%)	5 (16.1%)	
Obvious	42 (38.5%)	28 (35.9%)	14 (45.2%)	
*^M^MGMT status*				
Yes	37 (33.9%)	25 (32.1%)	12 (36.4%)	.508
No	72 (66.1%)	53 (68.0%)	19 (61.3%)	
*Diagnosis*				
TERTp-mutant	63	49	14	.092
TERTp-wildtype	46	29	17	

*Note*: Categorical variables were presented as the number (percentage). Age was presented as mean ± standard deviation.

M MGMT status = MGMT methylation status.

**Table 3:** Comparison of clinical characteristics according to *TERTp* mutant status in the training set and validation set.

Characteristics	Training set (n = 78)			Validation set (n = 31)		
	TERTp-mutant (n = 49)	TERTp-wildtype (n = 29)	*P*-value	TERTp-mutant ( = 14)	TERTp-wildtype (n = 17)	*P*-value
*Gender*						
Male	29 (59.2%)	19 (65.5%)	.578	9 (64.3%)	10 (58.8%)	.756
Female	20 (40.8%)	10 (34.5%)		5 (35.7%)	7 (41.2%)	
Age (year)	53.6 (11.3)	44.7 (16.4)	.083	52.4 (11.8)	43.4 (12.9)	.191
*Midline*						
Involving	8 (16.3%)	5 (17.2%)	.917	1 (7.1%)	5 (29.4%)	.118
Not involving	41 (83.7%)	24 (82.8%)		13 (92.9%)	12 (70.6%)	
*Laterality*						
Unilateral	41 (83.7%)	21 (72.4%)	.234	11 (78.6%)	13 (76.5%)	.889
Bilateral	8 (16.3%)	8 (27.6%)		3 (21.4%)	4 (23.5%)	
*Peritumoral edema*						
Enhancing margin	33 (67.4%)	15 (51.7%)	.171	7 (50.0%)	9 (52.9%)	.871
Poorly defined	16 (32.7%)	14 (48.3%)		7 (50.0%)	8 (47.1%)	
*Enhancing margin*						
Well defined	21 (57.1%)	13 (44.8%)	.865	7 (50.0%)	12 (70.6%)	.241
Poorly defined	28 (42.9%)	16 (55.2%)		7 (50.0%)	5 (29.4%)	
*Enhancement style*						
No	14 (28.6%)	6 (20.7%)	.165	8 (57.1%)	4 (23.5%)	.217
Irregular-enhancement	21 (42.9%)	14 (48.3%)		3 (21.4%)	5 (29.4%)	
Ring-enhancement	12 (24.5%)	4 (13.8%)		1 (7.1%)	5 (29.4%)	
Nodular-enhancement	2 (4.1%)	5 (17.2%)		2 (14.3%)	3 (17.7%)	
*Enhancement extent*						
No	14 (28.6%)	6 (20.7%)	.742	8 (57.1%)	4 (23.5%)	.146
Slight	18 (36.7%)	12 (41.4%)		2 (14.3%)	3 (17.7%)	
Obvious	17 (34.7%)	11 (37.9%)		4 (28.6%)	10 (58.8%)	
*^M^MGMT status*						
Yes	18 (36.7%)	7 (24.1%)	.249	5 (35.7%)	7 (41.2%)	.571
No	31 (63.3%)	22 (75.9%)		9 (64.3%)	10 (58.8%)	

*Note*: Categorical variables were presented as the number (percentage). Age was presented as mean ± standard deviation.

M MGMT status = MGMT methylation status.

### 3.2. Radiomic feature selection, model construction, and validation

**Figure 2.**
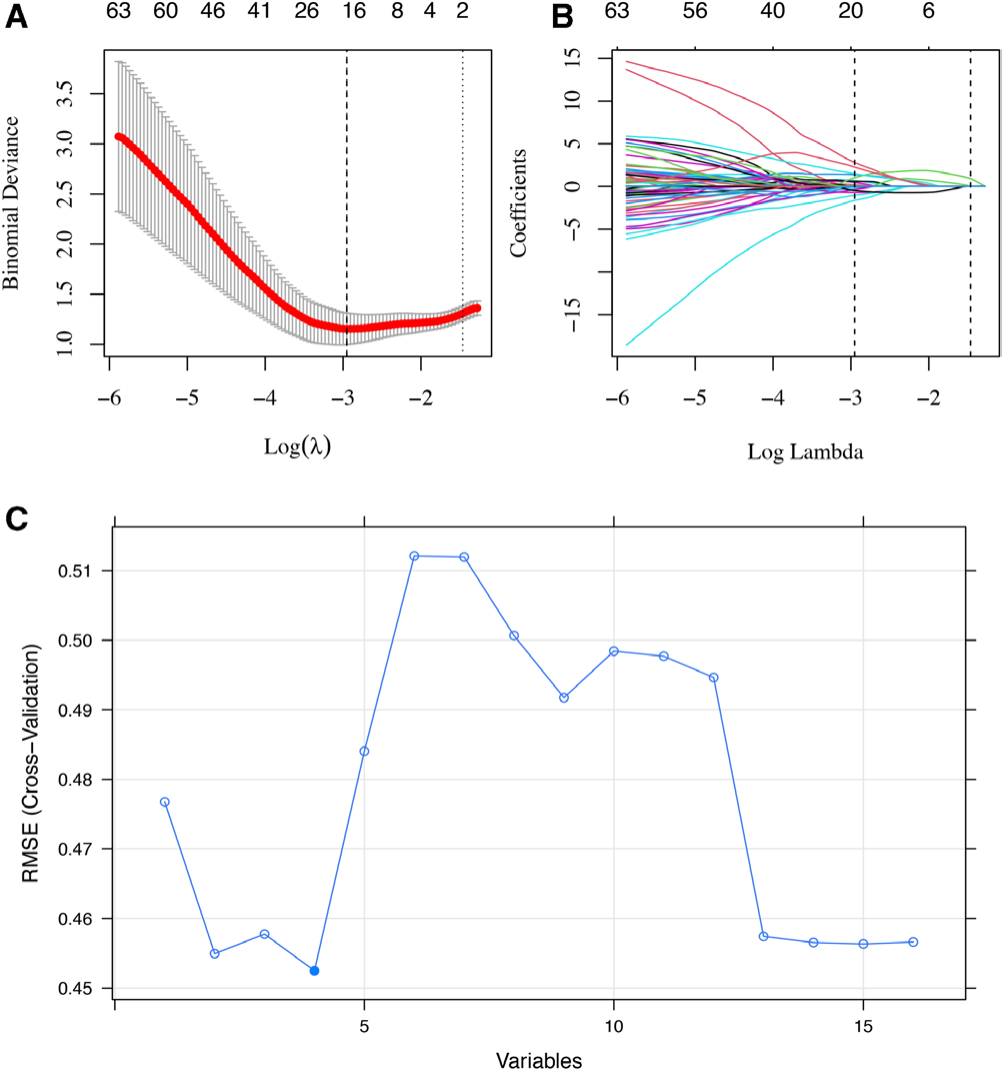
Process of radiomic feature extraction. (A) Parameter (λ) selection process with the 10-fold cross-validation via minimum error criterion by elastic net algorithm (alpha = 0.9). The optimal values are indicated by the dotted vertical lines (the left 1), and the value was 0.0522. (B) Elastic net coefficient profiles of the 868 radiomics features with selected log λ value and 10-fold cross-validation. Sixteen radiomic features with nonzero coefficients were chosen. (C) Final 4 features were confirmed by recursive feature elimination algorithm.

In total, we extracted 1304 radiomic features from 1 sequence of 1 patient with the Pyradiomics algorithm (see Tables S2, Supplemental Digital Content, http://links.lww.com/MD/L64, S3, Supplemental Digital Content, http://links.lww.com/MD/L65, S4, Supplemental Digital Content, http://links.lww.com/MD/L66, and S5, Supplemental Digital Content, http://links.lww.com/MD/L67, which illustrates the radiomics features extracted from the sequences). Thus, total 2608 radiomic features were collected from the T2WI and CE-T1W1 series from 1 patient. And, Wilcoxon rank-sum test, elastic net, and the RFE were used to reduce the overfitting. At first, 868 radiomic features were selected by the Wilcoxon rank-sum test with a threshold of *P* value of .05. Then, as shown in Figure 2, the elastic net algorithm (alpha = 0.9, and λ = 0.0522) confirmed 16 representative radiomic features.

Ultimately, 4 radiomic features (1 texture features and 3 wavelet features) that gave the best performance were selected as final representative features based on the RFE algorithm with 5-fold cross validation (Fig. 2).

**Figure 3.**
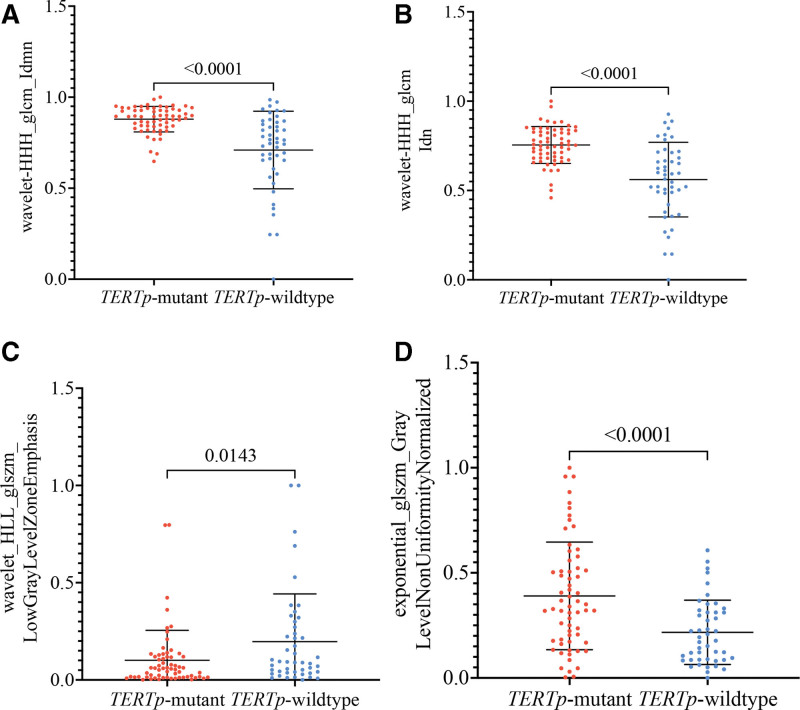
The final 4 radiomic features had significant differences between TERTp-mutant and TERTp-wildtype in IDHwt LGAs. (A) Wavelet-HHH_glcm_Idmn; (B) wavelet-HHH_glcm_Idn; (C) exponential_glszm_GrayLevelNonUniformityNormalized; (D) wavelet-HLL_glszm_LowGrayLevlZoneEmphasis.

**Figure 4.**
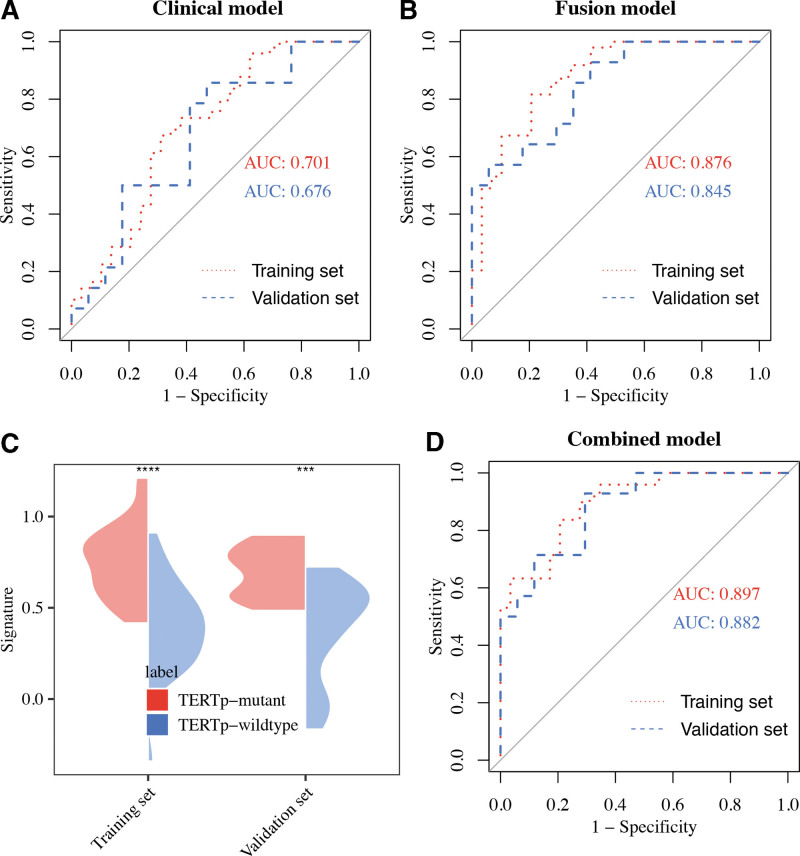
ROC curves for the fusion radiomic model (A), clinical model (B), and combined radiomic nomogram (D) in the training and validation sets. (C) The signature distribution of the fusion radiomic model between TERTp-mutant and TERTp-wildtype in IDHwt LGAs was shown as a violin plot.

Three features were selected from the T2WI images, and 1 from the CE-T1WI images. The 4 selected radiomic features have a significant difference between TERTp-mutant and TERTp-wildtype (*P* < .05; Fig. 3A–D, and Table 4). The 4 selected features were then entered into SVM to create a fusion radiomic model. And, the fusion radiomic model demonstrated favorable discrimination with AUC values of 0.876 and 0.845 in the training and validation sets, respectively (Fig. 4B). Furthermore, there were significant differences in the signature distributions of fusion radiomic models between TERTp-mutant and TERTp-wildtype patients in both training and validation sets (Fig. 4C).

**Table 4:** Detail information of the final 6 radiomic features.

*Sequence*	Feature name	Feature type	TERTp-mutant	TERTp-wildtype	*P*-value
T2WI	Wavelet-HHH_glcm_Idmn	Wavelet	0.879 ± 0.070	0.710 ± 0.213	.0001
	Wavelet-HHH_glcm_Idn	Wavelet	0.754 ± 0.103	0.561 ± 0.209	.0001
	exponential_glszm_GrayLevelNonUniformityNormalized	Texture	0.390 ± 0.256	0.217 ± 0.153	.0001
CET1	Wavelet-HLL_glszm_LowGrayLevlZoneEmphasis	Wavelet	0.101 ± 0.154	0.197 ± 0.246	.0143

*Note*: The values in the table are mean ± sample error of the mean.

CE-T1WI = contrast-enhanced T1-weighted imaging; T2WI = T2-weighted imaging.

**Figure 5.**
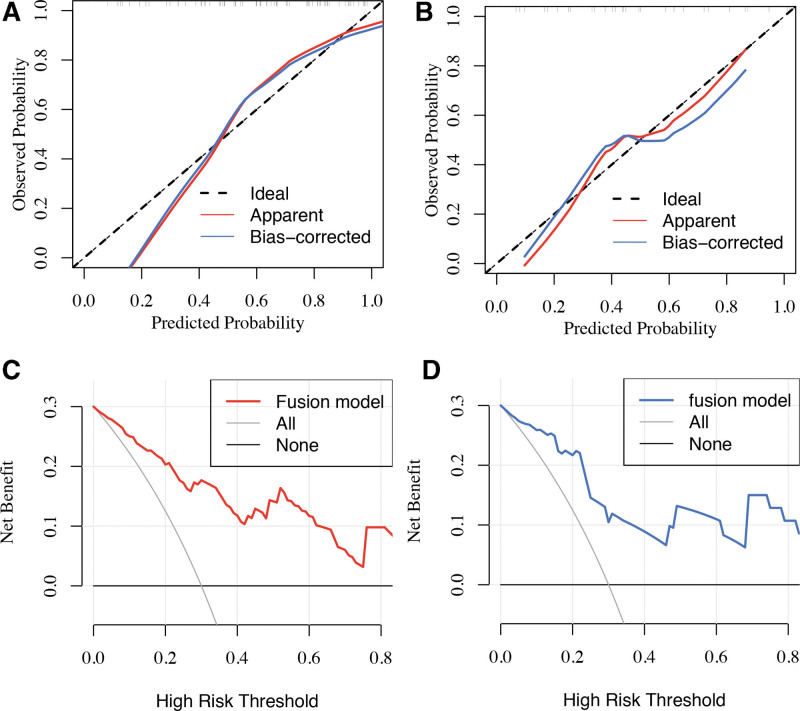
Calibration curves and decision curves of the fusion radiomic model in trading set (A and C) and validation set (B and D). (A and C) Calibration curve demonstrates the agreement between actual observations and predictions of the model in terms of TERTp-mutant diagnosis. *Y*-axis indicates the actual rate. *X*-axis indicates the predicted probability. A 45° black line represents the perfect match between the actual rate and the predicted probabilities. The performance of the fusion radiomic model was shown as the red (apparent) and blue (bias corrected) lines, which fit to the black line closer and the prediction was better. (B and D) *Y*-axis represents the net benefit. The model was shown in the red (training set) and blue (validation set) line. The gray line and the black line represent the assumption that all patients were classified as TERTp-mutant and TERTp-wildtype, respectively.

Additionally, the calibration curves and Hosmer–Lemeshow tests showed good agreement between observations and predictions in the training set (*P* = .5; Fig. 5A) and the validation set (*P* = .7; Fig. 5B). Figure 5C and D illustrate the DCA curves of the fusion radiomic model. There was clearly a net benefit from the fusion radiomic model over the other 2 schemes, and training and validation sets have threshold probabilities of >0% and >0%, respectively. Based on these results, it appears that the radiomic fusion model is clinically useful.

### 3.3. Combined model construction and validation

Based on multivariable logistic regression analysis, a clinical model was built using the 9 clinical features in the training set. In the validation set, we verified the performance of this model. According to Figure 4A, the AUC values for training and validation were 0.701 and 0.676, respectively.

**Figure 6.**
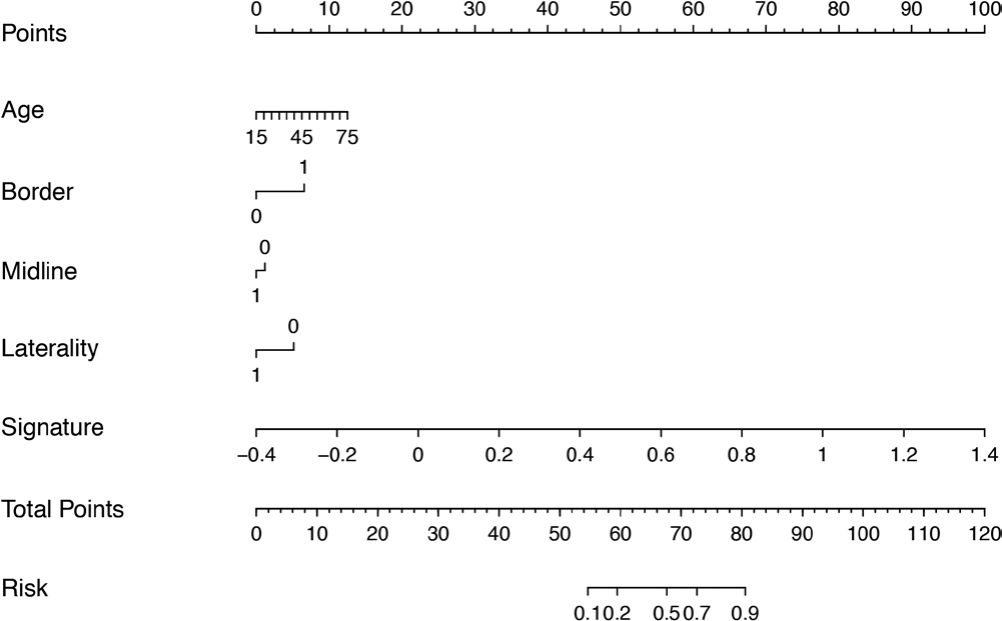
A nomogram of the combined radiomic model.

**Figure 7.**
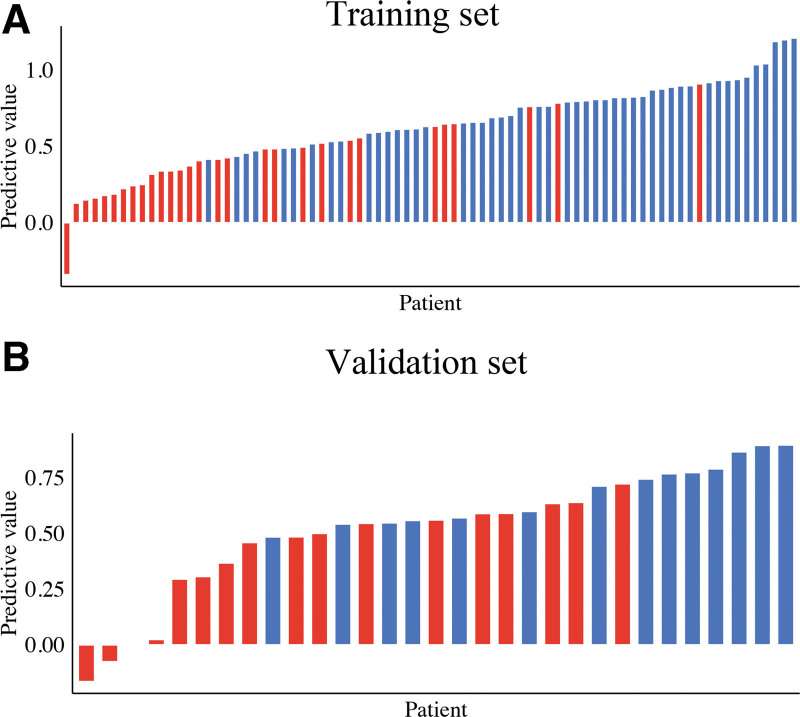
Bar plots of the combined radiomic nomogram in the training set (A) and validation set (B). Patients with the correct diagnosis of the combined radiomic nomogram are indicated by the blue and green histograms above and below the horizontal axis, respectively.

Then, 4 clinical characteristics (including age, enhancing border, laterality, and midline) were identified by AIC. In addition, the above clinical characteristic and the signature of fusion radiomic model were used to construct the combined radiomic model, and the AUC value of the nomogram was 0.897 in the training set and 0.882 in the validation set (Fig. 4D). In addition, the predictive ACC of the model in the training set was 0.821, while predictive ACC in the validation set was 0.806 (Table 5). And, the model was presented as nomogram in Figure 6. The ACC of the model in the training set and the validation set was shown in bar plots (Fig. 7).

**Table 5:** Detailed information about the diagnostic ability of the 3 models.

Model	Performance	AUC	ACC	Sensitivity	Specificity	PPV	NPV
Fusion model	Training set	0.876	0.795	0.796	0.793	0.870	0.697
	Validation set	0.845	0.710	0.857	0.588	0.632	0.846
Clinical model	Training set	0.701	0.701	0.736	0.647	0.766	0.615
	Validation set	0.676	0.679	0.673	0.690	0.786	0.553
Combined model	Training set	0.897	0.821	0.837	0.793	0.875	0.889
	Validation set	0.882	0.806	0.929	0.706	0.733	0.929

ACC = accuracy, AUC = area under curve, NPV = negative predictive value, PPV = positive predict value.

**Figure 8.**
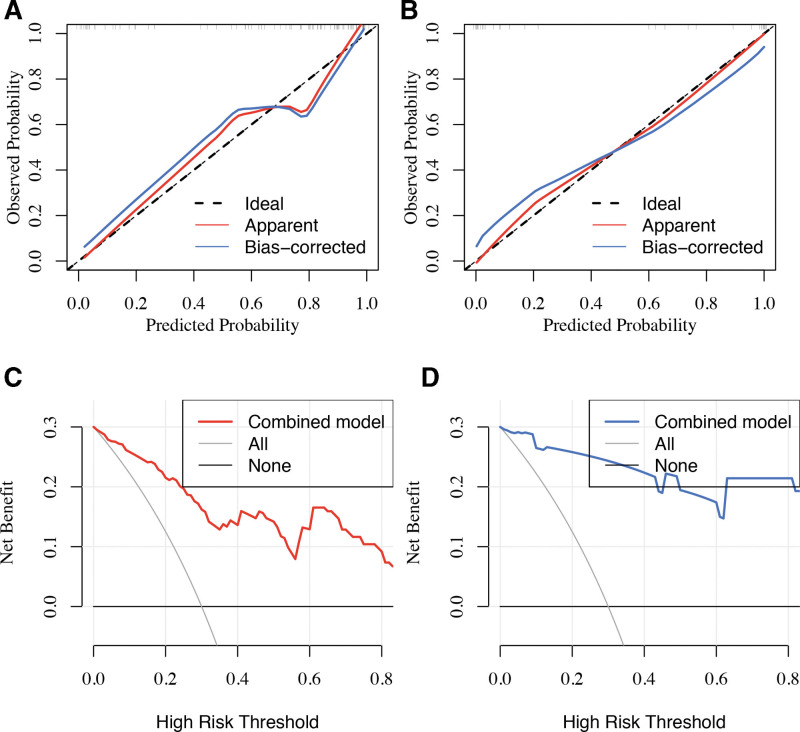
Calibration curves and decision curves of the combined radiomic nomogram in trading set (A and C) and validation set (B and D). (A and C) Calibration curve demonstrates the agreement between actual observations and predictions of the model in terms of TERTp-mutant diagnosis. *Y*-axis indicates the actual rate. *X*-axis indicates the predicted probability. A 45° black line represents the perfect match between the actual rate and the predicted probabilities. The performance of the combined radiomic nomogram was shown as the red (apparent) and blue (Bias corrected) lines, which fit to the black line closer and the prediction was better. (B and D) *Y*-axis represents the net benefit. The model was shown in the red (training set) and blue (validation set) line. The gray line and the black line represent the assumption that all patients were classified as TERTp-mutant and TERTp-wildtype, respectively.

As shown in Figure 8, the calibration curves and Hosmer–Lemeshow tests revealed that the clinical radiomic nomogram has good agreement between observations and predictions in training set (*P* = .4; Fig. 8A) and validation set (*P* = .07; Fig. 8B). In addition, DCA curves identified that the combined model demonstrated a higher net benefit than the other 2 schemes, and the threshold probability of training set was >0% (Fig. 8C) and validation set was >0% (Fig. 8D). In conclusion, the results revealed that the combined radiomic nomogram has a significant clinical usefulness. Finally, the decision curve demonstrated that the combined radiomic nomogram has better performance than the clinical application. Furthermore, after the DeLong test, we found that the *P* value between the combined radiomic nomogram and clinical model was the smallest among the 3 models contrast although it was not <0.05 in the validation set. That indicated that clinical radiomic nomogram has a significant differentiation of TERTp mutation than the clinical model (Table 6).

**Table 6:** Comparison of the prediction with the combined radiomics nomogram, clinical, and radiomics model.

Model	Model 1	Model 2	*P* value
Training	Combined	Clinical	.009
	Combined	Fusion	.300
	Clinical	Fusion	.030
Validation	Combined	Clinical	.080
	Combined	Fusion	.400
	Clinical	Fusion	.200

## 4. Discussion

In our research, we delved deeper into radiological images of IDHwt LGAs by extracting 1304 features from CE-T1WI and T2WI sequences. This allowed us to mine the data more thoroughly, specifically in regards to the TERTp-mutation and TERTp-wildtype patients. Our radiomics signature model showed impressive performance in predicting the TERTp mutation status. To create a more comprehensive tool, we combined the radiomics model with clinical risk factors such as age, border, tumor crossing the midline, and laterality, constructing a personalized nomogram for predicting TERTp mutation status in IDHwt LGAs. We tested the model on an entirely new test cohort to ensure its generalizability, and the results demonstrated its efficiency. Overall, our combined model was the best in terms of overall net efficiency, as determined by decision curve analysis.

Traditionally, the grading of central nervous system tumors has relied heavily on their histological features. However, the addition of molecular markers as grading biomarkers has provided valuable prognostic information for various tumor types. Radiomics, on the other hand, uses advanced computing methods to extract potential features from radiological images, allowing for noninvasive and more comprehensive evaluation of tumors. Recent studies have demonstrated the usefulness of radiomics in predicting molecular subgroups of glioma, such as IDH mutations,^[6,18]^ TP53,^[19]^ and MGMT promoter methylations,^[20]^ as well as the recurrence and pseudo progression of high-grade glioma.^[21]^ And, many studies have shown that IDHwt LGAs with TERTp-mutant status have been shown to have a poor prognosis compared to TERTp-wildtype patients and the presence alone is sufficient for the diagnosis of glioblastoma, IDH wt.^[4,22–24]^ However, prediction of TERTp-mutation before surgery and molecular testing can be challenging, as the image features on CT and MRI scans or the pathologically features may appear similar. Furthermore, obtaining molecular information can be time-consuming and expensive, or not possible in some cases, such as non-surgery cases or inaccessible tumor specimens. Therefore, developing a noninvasive method to predict TERTp mutation status in IDHwt LGAs is both important and urgent.

Recent studies have demonstrated the potential of radiomics in predicting molecular markers in IDHwt LGAs. For instance, a study reported that radiomics could predict TERTp mutations and EGFR amplification with an AUC of 0.854, which increased to 0.863 after incorporating clinical information.^[11]^ In our study, the AUC of the radiomic model was slightly lower at 0.845, but the combined model outperformed the previous study with an AUC of 0.882. Additionally, the ACC values of the radiomic and combined models were 0.710 and 0.806, respectively. The calibration and decision curve analyses further supported the effectiveness of the combined model in discriminating TERTp mutation status. Another study investigated the use of radiomics extracted from dynamic [18F] FET PET to predict TERTp mutation status in glioblastoma,^[12]^ reporting an AUC of 0.82 and sensitivity of 0.92. Our study outperformed this study in terms of AUC and sensitivity, likely due to the inclusion of glioblastoma in that study and low grade IDHwt LGAs in our analysis. This suggests that the radiomic changes associated with TERTp mutations may differ between IDHwt LGAs and glioblastoma. Finally, a previous study that included both IDH mutant and IDH wildtype patients reported an AUC of 0.669 for predicting TERT promoter mutation status,^[25]^ which was inferior to our results. This may be attributed to the mixing of radiomic information from IDH mutant and IDH wildtype patients in the analysis.

To construct our classification model, we utilized 3 different algorithms to screen and select the most relevant radiomic features. This was essential to exclude irrelevant features that could potentially obscure important information and negatively impact the performance of the prediction model.^[8]^ Ultimately, we identified 4 features that were most significant in predicting the TERTp mutation status, with 3 features extracted from the CE-T1WI sequence and 1 from the T2WI sequence. The CE-T1WI sequence is highly effective in revealing the activity and necrotic areas of the tumor, while the T2WI sequence can reflect the anatomical location and cellularity of the tumor.^[26]^ Unlike previous studies that utilized advanced imaging techniques such as dynamic [18F] FET PET,^[12]^ our study focused on commonly used imaging sequences in most hospitals.^[27]^ This makes our results more widely applicable and accessible to a broader range of medical professionals. Furthermore, the combination of the CE-T1WI and T2WI sequences provides a more comprehensive evaluation of tumors, as it captures and reflects information from different aspects of the tumor. Interestingly, all the 4 selected features were texture-based. Texture analysis is a mathematical method used to quantify the spatial variations of gray-levels within an image to derive textural features. These textural features reflect intra-tumoral heterogeneity, and have been shown to be useful in predicting specific genetic mutations in patients with LGAs.^[28]^ This highlights the potential of texture-based radiomics features in enhancing the ACC of molecular subtyping and improving prognostic prediction in patients with brain tumors.^[11,29,30]^

Despite the promising results, the study has some limitations. Firstly, the sample size was relatively small and collected from a single center. The inclusion of more patients from multiple institutions could increase the robustness and generalizability of the combined radiomic model. Secondly, the process of manually delineating the ROI was time-consuming and subject to inter-observer variability. The use of semiautomatic or automatic delineation methods may improve the efficiency and consistency of the ROI segmentation.^[31]^ Additionally, there is currently no standardized method for tumor segmentation, and the tumor edge may not always be accurately determined on medical images, leading to potential measurement errors. Thirdly, the machine learning algorithm used in the study was limited to SVM, and other algorithms, for example, the enhanced KNN algorithm,^[32]^ could be explored in future studies to improve the performance of the radiomics model. Lastly, the radiomic nomogram developed in this study may serve as a complementary tool in clinical practice, but further research is needed to investigate whether radiomics can predict other clinically relevant factors, such as the WHO grade of gliomas with specific genetic mutations, such as *TERTp* mutation.

## 5. Conclusion

In this study, based on the image features of 2 sequences of T2W and CE-T1WI, a fusion radiomic model and a combined radiomic nomogram were constructed and have good ACC in diagnosis TERTp mutant status in IDHwt LGAs. Furthermore, the combined radiomic nomogram has the best performance and good clinical application.

## Acknowledgments

This study was supported by the National Natural Science Foundation of China (grant number 62027813, 2022YFE0112500, and 8180100922). The authors declare that there was no any interest for any person or organization. And, we acknowledged Yanghua Fan for assistance with data-handling.

## Author contributions

**Conceptualization:** Xulei Huo, Qiang Ji, Zhen Wu.

**Data curation:** Xulei Huo, Sihan Ma, Sipeng Zhu, Zhen Wu.

**Funding acquisition:** Ke Wang, Wenbin Li.

**Investigation:** Wenbin Li.

**Methodology:** Yali Wang, Wenbin Li.

**Resources:** Zhen Wu.

**Software:** Zhen Wu, Wenbin Li.

**Supervision:** Yali Wang, Feng Chen, Liang Wang, Wenbin Li.

**Validation:** Yali Wang, Zhen Wu.

**Writing – original draft:** Xulei Huo.

**Writing – review & editing:** Xulei Huo.

## Supplementary Material











## References

[1] Louis DN Wesseling P Aldape K. cIMPACT-NOW update 6: new entity and diagnostic principle recommendations of the cIMPACT-Utrecht meeting on future CNS tumor classification and grading. Brain Pathol. 2020;30:844–56. 32307792 10.1111/bpa.12832 PMC8018152

[2] Aibaidula A Chan AK Shi Z. Adult IDH wild-type lower-grade gliomas should be further stratified. Neuro Oncol. 2017;19:1327–37. 28575485 10.1093/neuonc/nox078 PMC5596181

[3] Wijnenga MMJ Dubbink HJ French PJ. Molecular and clinical heterogeneity of adult diffuse low-grade IDH wild-type gliomas: assessment of TERT promoter mutation and chromosome 7 and 10 copy number status allows superior prognostic stratification. Acta Neuropathol. 2017;134:957–9. 29052002 10.1007/s00401-017-1781-z

[4] Komori T. Grading of adult diffuse gliomas according to the 2021 WHO classification of tumors of the central nervous system. Lab Invest. 2022;102:126–33. 34504304 10.1038/s41374-021-00667-6

[5] Louis DN Perry A Wesseling P. 2021 WHO classification of tumors of the central nervous system: a summary. Neuro Oncol. 2021;23:1231–51. 34185076 10.1093/neuonc/noab106 PMC8328013

[6] Li Y Ammari S Lawrance L. Radiomics-based method for predicting the glioma subtype as defined by tumor grade, IDH mutation, and 1p/19q codeletion. Cancers (Basel). 2022;14:1–26. 10.3390/cancers14071778 PMC8997070 35406550

[7] Fan Y Liu P Li Y. Non-Invasive preoperative imaging differential diagnosis of intracranial hemangiopericytoma and angiomatous meningioma: a novel developed and validated multiparametric MRI-based clini-radiomic model. Front Oncol. 2021;11:792521. 35059316 10.3389/fonc.2021.792521 PMC8763962

[8] Fan Y Huo X Li X. Non-invasive preoperative imaging differential diagnosis of pineal region tumor: a novel developed and validated multiparametric MRI-based clinicoradiomic model. Radiother Oncol. 2022;167:277–84. 35033600 10.1016/j.radonc.2022.01.005

[9] Li G Li L Li Y. An MRI radiomics approach to predict survival and tumour-infiltrating macrophages in gliomas. Brain. 2022;145:1151–61. 35136934 10.1093/brain/awab340 PMC9050568

[10] Kihira S Derakhshani A Leung M. Multi-parametric radiomic model to predict 1p/19q co-deletion in patients with IDH-1 mutant glioma: added value to the T2-FLAIR mismatch sign. Cancers (Basel). 2023;15:1037. 36831380 10.3390/cancers15041037 PMC9954034

[11] Park CJ Han K Kim H. MRI features may predict molecular features of glioblastoma in isocitrate dehydrogenase wild-type lower-grade gliomas. AJNR Am J Neuroradiol. 2021;42:448–56. 33509914 10.3174/ajnr.A6983 PMC7959428

[12] Li Z Kaiser L Holzgreve A. Prediction of TERTp-mutation status in IDH-wildtype high-grade gliomas using pre-treatment dynamic [(18)F]FET PET radiomics. Eur J Nucl Med Mol Imaging. 2021;48:4415–25. 34490493 10.1007/s00259-021-05526-6 PMC8566644

[13] van Griethuysen JJM Fedorov A Parmar C. Computational radiomics system to decode the radiographic phenotype. Cancer Res. 2017;77:e104–7. 29092951 10.1158/0008-5472.CAN-17-0339 PMC5672828

[14] Park BE Jang WS Yoo SK. Texture analysis of supraspinatus ultrasound image for computer aided diagnostic system. Healthc Inform Res. 2016;22:299–304. 27895962 10.4258/hir.2016.22.4.299 PMC5116542

[15] Qu J Shen C Qin J. The MR radiomic signature can predict preoperative lymph node metastasis in patients with esophageal cancer. Eur Radiol. 2019;29:906–14. 30039220 10.1007/s00330-018-5583-z

[16] Guyon I Weston J Barnhill S. Gene selection for cancer classification using support vector machines. Mach Learn. 2002;46:389–422.

[17] Hearst MA Dumais ST Osuna E. Support vector machines. IEEE Intelligent Systems and their applications. 1998;13:18–28.

[18] Kandalgaonkar P Sahu A Saju AC. Predicting IDH subtype of grade 4 astrocytoma and glioblastoma from tumor radiomic patterns extracted from multiparametric magnetic resonance images using a machine learning approach. Front Oncol. 2022;12:879376. 36276136 10.3389/fonc.2022.879376 PMC9585657

[19] Zhang X Tian Q Wang L. Radiomics strategy for molecular subtype stratification of lower-grade glioma: detecting IDH and TP53 mutations based on multimodal MRI. J Magn Reson Imaging. 2018;48:916–26. 29394005 10.1002/jmri.25960

[20] Chen S Xu Y Ye M. Predicting MGMT promoter methylation in diffuse gliomas using deep learning with radiomics. J Clin Med. 2022;11:1–11. 10.3390/jcm11123445 PMC9224690 35743511

[21] Jing H Yang F Peng K. Multimodal MRI-based radiomic nomogram for the early differentiation of recurrence and pseudoprogression of high-grade glioma. Biomed Res Int. 2022;2022:4667117. 36246986 10.1155/2022/4667117 PMC9553483

[22] Bale TA Jordan JT Rapalino O. Financially effective test algorithm to identify an aggressive, EGFR-amplified variant of IDH-wildtype, lower-grade diffuse glioma. Neuro Oncol. 2019;21:596–605. 30496526 10.1093/neuonc/noy201 PMC6502496

[23] Fujimoto K Arita H Satomi K. TERT promoter mutation status is necessary and sufficient to diagnose IDH-wildtype diffuse astrocytic glioma with molecular features of glioblastoma. Acta Neuropathol. 2021;142:323–38. 34148105 10.1007/s00401-021-02337-9

[24] Tesileanu CMS Dirven L Wijnenga MMJ. Survival of diffuse astrocytic glioma, IDH1/2 wildtype, with molecular features of glioblastoma, WHO grade IV: a confirmation of the cIMPACT-NOW criteria. Neuro Oncol. 2020;22:515–23. 31637414 10.1093/neuonc/noz200 PMC7158657

[25] Yan J Zhang B Zhang S. Quantitative MRI-based radiomics for noninvasively predicting molecular subtypes and survival in glioma patients. npj Precis Oncol. 2021;5:72. 34312469 10.1038/s41698-021-00205-z PMC8313682

[26] Tan Y Mu W Wang XC. Improving survival prediction of high-grade glioma via machine learning techniques based on MRI radiomic, genetic and clinical risk factors. Eur J Radiol. 2019;120:108609. 31606714 10.1016/j.ejrad.2019.07.010

[27] Strauss SB Meng A Ebani EJ. Imaging glioblastoma posttreatment: progression, pseudoprogression, pseudoresponse, radiation necrosis. Neuroimaging Clin N Am. 2021;31:103–20. 33220823 10.1016/j.nic.2020.09.010

[28] Molina D Perez-Beteta J Martinez-Gonzalez A. Influence of gray level and space discretization on brain tumor heterogeneity measures obtained from magnetic resonance images. Comput Biol Med. 2016;78:49–57. 27658261 10.1016/j.compbiomed.2016.09.011

[29] Li Y Liu X Xu K. MRI features can predict EGFR expression in lower grade gliomas: A voxel-based radiomic analysis. Eur Radiol. 2018;28:356–62. 28755054 10.1007/s00330-017-4964-z

[30] Li Y Qian Z Xu K. Radiomic features predict Ki-67 expression level and survival in lower grade gliomas. J Neurooncol. 2017;135:317–24. 28900812 10.1007/s11060-017-2576-8

[31] Zhou Z Xiong Z Cheng R. Volumetric visceral fat machine learning phenotype on CT for differential diagnosis of inflammatory bowel disease. Eur Radiol. 2022;33:1862–72. 36255487 10.1007/s00330-022-09171-x

[32] Ng SP Khor YP Lim HK. Fabrication of concentrated palm olein-based diacylglycerol oil-soybean oil blend oil-in-water emulsion: in-depth study of the rheological properties and storage stability. Foods. 2020;9:877. 32635372 10.3390/foods9070877 PMC7404400

